# A novel class of chemicals that react with abasic sites in DNA and specifically kill B cell cancers

**DOI:** 10.1371/journal.pone.0185010

**Published:** 2017-09-19

**Authors:** Shanqiao Wei, Madusha L. W. Perera, Ramin Sakhtemani, Ashok S. Bhagwat

**Affiliations:** 1 Department of Chemistry, Wayne State University, Detroit, Michigan, United States of America; 2 Immunology and Microbiology, Wayne State University, Detroit, Michigan, United States of America; University of South Alabama Mitchell Cancer Institute, UNITED STATES

## Abstract

Most B cell cancers overexpress the enzyme activation-induced deaminase at high levels and this enzyme converts cytosines in DNA to uracil. The constitutive expression of this enzyme in these cells greatly increases the uracil content of their genomes. We show here that these genomes also contain high levels of abasic sites presumably created during the repair of uracils through base-excision repair. We further show that three alkoxyamines with an alkyne functional group covalently link to abasic sites in DNA and kill immortalized cell lines created from B cell lymphomas, but not other cancers. They also do not kill normal B cells. Treatment of cancer cells with one of these chemicals causes strand breaks, and the sensitivity of the cells to this chemical depends on the ability of the cells to go through the S phase. However, other alkoxyamines that also link to abasic sites- but lack the alkyne functionality- do not kill cells from B cell lymphomas. This shows that the ability of alkoxyamines to covalently link to abasic sites is insufficient for their cytotoxicity and that the alkyne functionality may play a role in it. These chemicals violate the commonly accepted bioorthogonality of alkynes and are attractive prototypes for anti-B cell cancer agents.

## Introduction

The enzyme activation-induced deaminase (AID) is expressed at high levels in B lymphocytes during their normal development following an infection, and converts cytosines in DNA to uracil [1–5]. Processing of this rare DNA base by the cells creates targeted mutations and deletions in the immunoglobulin genes. These genetic alterations increase the affinity of antibodies for antigens through mutations, and cause isotype switching within the antibody proteins. These phenomena are respectively referred to as somatic hypermutation and class-switch recombination [6–9]. While most B cells complete their developmental program and down-regulate AID prior to leaving the site of their development, germinal centers, some cells continue to express AID at high levels outside germinal centers. This causes genetic alterations including mutations outside the immunoglobulin loci and chromosome translocations [10, 11]. This sometimes results in malignant cellular transformation and this explains the strong correlation between B cell cancers of germinal center origin and high-level expression of AID [12–16].

Many B cell tumors and tumor-derived cell lines also contain highly elevated levels of uracils in their genomes that correlate with AID expression [17, 18]. In different studies, cell lines derived from non-Hodgkin B cell lymphomas or leukemias (B-NHLs) were found to contain ~80- to 120-fold [17] or ~4- to 30-fold [18] higher levels of genomic uracils compared to normal circulating B cells. B-NHL patient tumors showed a wider range of uracil levels ranging from normal levels to 120-fold higher than normal levels [17]. Again, the higher uracil levels in these cells were correlated with higher levels of AID expression in tumor cells [17, 18].

Uracils in mammalian genomes are removed by the nuclear form of the uracil-DNA glycosylase, UNG2 [19–22], and the resulting abasic sites (a.k.a. apurinic/apyrimidinic or AP sites) are repaired through the base excision repair pathway (BER pathway, S1 Fig). UNG2 is an efficient enzyme with a high turnover rate [23], and hence we hypothesized that most of the uracils created by AID in B-NHL genomes should be removed by UNG2 creating AP sites. Furthermore, we speculated that if these AP sites were not quickly repaired by BER, they would accumulate in B-NHL genomes and cause cell death (S1 Fig).

In this study, we show that human B-NHL cell lines with high AID levels indeed contain elevated levels of AP sites, while none of the cancer cell lines derived from other tissues have high AP site levels. Furthermore, we show that a class of chemicals that covalently links to AP sites also kills B-NHL cells, but not normal human cells or other cancer cells. We define below the chemical functionalities required for such specific killing of cancer cells and discuss the likely mechanism underlying the lethal action of these chemicals.

## Materials and methods

### Cell lines and primary human B cells

The human cell lines HeLa (cervical cancer), MCF-7 and MDA-MB-453 (breast cancer), HEK293T (immortalized embryonic kidney), Raji and Daudi (Burkitt lymphoma) were obtained from American Type Culture Collection (ATCC). The human lung cancer cell line A549 was kindly provided by Dr. Young-Hoon Ahn (Wayne State University). The Burkitt lymphoma cell line Ramos 1 was obtained from Dr. Alberto Martin (University of Toronto). The primary human epidermal keratinocytes (HEKn) were obtained from Thermo-Fisher. All B-cell lymphoma cell lines were cultured in RPMI-1640 media (Sigma-Aldrich) supplemented with 10% fetal bovine serum (FBS) (HyClone) and 1% penicillin-streptomycin (Pen-Strep; Thermo-Fisher). HeLa, A549 and HEK293T cells were cultured in DMEM medium (HyClone) with 10% FBS and 1% Pen-Strep. MCF-7 cells were grown in MEM medium (HyClone) supplemented with10% FBS and 1% Pen-Strep, while MDA-MB-453 cells were grown in Leibovitz's L-15 medium supplemented with10% FBS and 1% Pen-Strep. All cultures were incubated at 37°C in a humidified incubator with 5% CO_2_ in T-25 cm^2^ or T-75 cm^2^ flasks; or 48-well plates (CytoOne, USA scientific). All the cell lines were used within 12 months of their validation.

American Red Cross blood donation center (Livonia, MI) collected blood from healthy volunteer donors who had consented to the use of the blood in research and deidentified the samples. Dr. Martin Bluth (Associate Director of the Transfusion Service, Harper University Hospital, Detroit Medical Center) obtained this blood in apheresis cones and kindly provided it to us without any personal identification information. It was exempted from IRB review because the samples “cannot be identified, directly or through identifiers linked to the subjects” (exception #4). Peripheral blood mononuclear cells (PBMC) were isolated from these blood samples with Ficoll-Paque Premium density- gradient media (Stem Cell Technologies). The purified B cells were isolated from PBMC with Easy Sep^™^ direct human B cell isolation kit (Stem Cell Technologies). Purified B cells were cultured in RPMI-1640 media supplemented with 10% FBS and 1% Pen-Strep and activated using 1 μg/mL anti-CD-40 antibody (Peprotech) and 50 ng/mL IL-4 (Peprotech).

### Genomic DNA isolation

The human cells were harvested by centrifugation and lysed by incubation for 1hr at 37°C in Tris-EDTA buffer (TE; pH 7.8) containing 2 μg/mL of RNase A and 0.5% SDS, followed by incubation with Proteinase K (100 μg /ml, Qiagen) at 56°C for 3 hours. The DNA was isolated by phenol/chloroform extraction and ethanol precipitation and dissolved in TE.

### Labeling and quantification of AP sites

It should be noted that our assays are expected to detect all aldehydic lesions in DNA. These include intact AP sites, AP sites cleaved by an AP endonuclease or other enzymes, the formamido forms of a number of oxidation and alkylation-induced adducts [24], and we use the phrase “AP site quantification” for textual convenience. Quantification of AP sites using AA3 was performed as described previously [24]. Briefly, freshly prepared genomic DNA was fragmented using the restriction enzyme HaeIII (New England Biolabs), the AP sites were reacted with AA3 and the AA3-tagged DNA was conjugated to Cy5 using the click reaction. Cy5 azide (Lumiprobe) was added to a solution of AA3-linked DNA to 0.5 mM followed by the addition of a freshly prepared solution of CuBr/TBTA (1:5 in DMSO/t-BuOH 3:1, 0.5 mM, Sigma-Aldrich). The mixture was shaken at 45°C for 1 hour. The Cy5-labeled DNA was heated at 95°C for 5 minutes prior to being transferred to a positively charged nylon membrane. The membrane was scanned using a Typhoon 9210 phosphorimager (GE Healthcare). The images were analyzed using ImageQuant software. In some cases, the Cy5 fluorescence was directly measured using Synergy H1 Hybrid Reader (BioTEK) and the fluorescence intensities were obtained directly from the instrument.

The protocol for labeling AP sites in DNA using Aldehyde-reactive probe (ARP; Dojindo Molecular Technologies) or AA3 has been described previously [17, 24]. Briefly, ARP was added to the solution of genomic DNA to 2 mM and the mixture was incubated at 37°C for 30 minutes. In parallel, a 75 base-pair duplex DNA with one uracil (5'-T_37_UT_37_-3'/5'-A_37_GA_37_-3') was treated with UDG to create AP sites and was also treated with ARP or AA3. This served as an AP site standard. All DNAs were purified by phenol/chloroform extraction and ethanol precipitation, followed by passage through a MicroSpin G-25 column (GE Healthcare). The DNA was immobilized on a nylon membrane and bound to Cy5-Streptavidin (GE Healthcare) and the Cy5 fluorescence was quantified as described above.

### Quantification of expression of BER genes using RT-PCR

Total RNA was extracted from Daudi, Raji and HeLa cells using TRIzol reagent (ThermoFisher Scientific) according to manufacturer’s instructions. The cDNA was synthesized by ProtoScript^®^ II First Strand cDNA Synthesis Kit (New England Biolabs) using the Oligo d(T)_23_ as primers. The mRNA expression levels were determined using primers listed in S1 Table and PowerUP SYBR Green Mix (Applied Biosystems) according to the manufacturer’s protocol. Four replicate reactions were carried out in 20 μL volume. The 7500 Fast Real-Time PCR Systems (Applied Biosystems) was used to perform the RT-PCR expression measurements.

The Ct values were normalized to β-actin gene (ACT B) and the gene expression was calculated using the equation- $Expression\;level\;of\;gene\;X=2{-}^{\left(Ct_{\left(x\right)}-{\bar{Ct}}_{\left(ACTB\right)}\right)}$. GraphPad Prism software was used to plot the data.

### Quantification of AP sites tagged *in vivo* using AA3

Daudi cells were grown in appropriate growth media and treated with 5 mM AA3 for indicated lengths of times. Genomic DNA was isolated and digested with HaeIII. The digested DNA was conjugated with Cy5 azide through click reaction prior to being transferred to a positively charged nylon membrane. The membrane was scanned and analyzed as described above.

To compare *in vivo* DNA binding by AA3 in dead and viable Daudi cells, the dead cells were separated using apoptotic cell isolation kit (PromoKine) following 5 hr AA3 treatment. Genomic DNAs were extracted from both types of cells, fragmented with HaeIII and labeled with Cy5 azide for quantification as described above.

### Cytotoxicity assays

B-cell lymphoma cell lines (Raji, Ramos 1, Daudi and Toledo) were seeded in 48-well tissue culture plates at 2x10^5^ to 6x10^5^ cells/mL. The cells were treated with an alkoxyamine at indicated concentrations and harvested after 24 hours. In some experiments, the Daudi cells were pre-treated with MX at 10 mM for 6 hours prior to overnight incubation with AA3 at 1 mM. Normal human B cells were stimulated to divide for three days and then seeded in 48-well plates at 2x10^5^ to 5x10^5^ cellsl/mL. They were treated with AA3 at indicated concentrations and harvested after 24 hours. HeLa, MCF-7, MDA-MB-453, A549, HEK293T and HEKn cells were seeded in 48-well tissue culture plates at 3x10^4^ to 6x10^4^ cell/mL and grown overnight. The following day, the cells were treated with AA3 at indicated concentrations and harvested after 24 hours. In all cases, the viability of cells was determined by the trypan blue exclusion assay [25].

To test the toxicity of CRT0044876 (Sigma-Aldrich), Raji cells were seeded in 48-well tissue culture plates at 2x10^5^ to 6x10^5^ cells/mL. The cells were treated with CRT0044876 at indicated concentrations and harvested after 24 hours. We were unable to test concentrations of CRT0044876 above 1 mM because at higher concentrations of the compound precipitated when added to the cell growth media. HeLa cells were seeded in 48-well tissue culture plates at 3x10^4^ to 6x10^4^ cell/mL and grown overnight. The following day, the cells were treated with CRT0044876 at indicated concentrations and harvested after 24 hours. As a positive control, cells were treated with 5 mM AA3 and as a negative control cells were treated with 5 mM MX for 24 hours. In all cases, the viability of cells was determined by the trypan blue exclusion assay.

### Visualization and quantification of γH2AX foci

Daudi cells were treated with 5 mM AA3 for 5 hr or 50 μg/ml Phleomycin (Cayman Chemicals) for 24 hr and harvested by centrifugation. The cells from the pellets were resuspended in a hypotonic solution containing 75 mM KCl, incubated at 37°C for 15 minutes and centrifuged onto microscope slides using a StatSpin Cytofuge (Beckman Coulter). After fixation of cells in 4% paraformaldehyde in PBS, they were permeabilized with 0.3% Triton X-100 in PBS. Cells were then blocked with 10% Goat serum in PBS and incubated with 2 μg/mL anti-phospho-histone H2A.X (Ser139) antibody solution (Millipore). The slides were washed with PBS, followed by incubation with 1 μg/mL goat anti-mouse antibody conjugated with Cy3 (Thermo-Fisher). The slides were mounted with DAPI Mounting Medium and visualized using a fluorescence microscope (Nikon E800). The visual fields with multiple nuclei were photographed and Cy3 fluorescence per nucleus was quantified using the ImageJ software.

### Cell synchronization and treatment with AA3

The cells were treated with 100μM L-mimosine (Sigma-Aldrich) for 24 hr to arrest them in the G1 phase. Half of the mimosine-treated cells were harvested by centrifugation, resuspended in fresh growth media and cultured for additional 4 hr to allow cells reenter S phase. The remaining cells were maintained in mimosine-containing media for additional 4 hr. Both mimosine-treated cells and untreated controls were seeded into a 48-well plate and treated with AA3 at 5 mM for 5 hr. The viability of cells was determined by the Trypan blue exclusion assay.

To confirm that mimosine-treated cells were arrested in the G1 phase, the cells were stained with Propidium iodide (PI, Sigma-Aldrich) and analyzed by flow cytometry. Briefly, cells were harvested by centrifugation and cell pellets were washed with cold Dulbecco's Phosphate-Buffered Saline (DPBS, HyClone) and resuspended into 0.5 mL cold DPBS. Cold 70% ethanol (4 mL) was added drop-wise into the cell suspension to fix the cells. After incubation at 4°C for 1 hour, the cells were harvested by centrifugation, washed with cold DPBS and resuspended in 0.5 mL PI staining solution. After incubation at 37°C for 30 min, the cells were washed with cold DPBS and resuspended in cold DPBS. The cell samples were analyzed by flow cytotometry.

## Results

### High levels of abasic sites in B cell cancers

**Fig 1:**
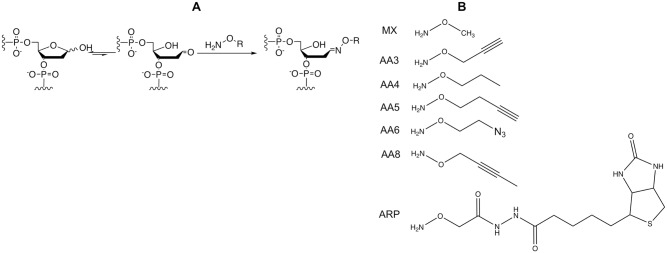
Reaction of alkoxyamines with AP sites. (A) The open-chain aldehyde form of an AP site in DNA reacts with an alkoxyamine (H_2_N-O-R). (B) Structures of different alkoxyamines used in this study.

**Fig 2:**
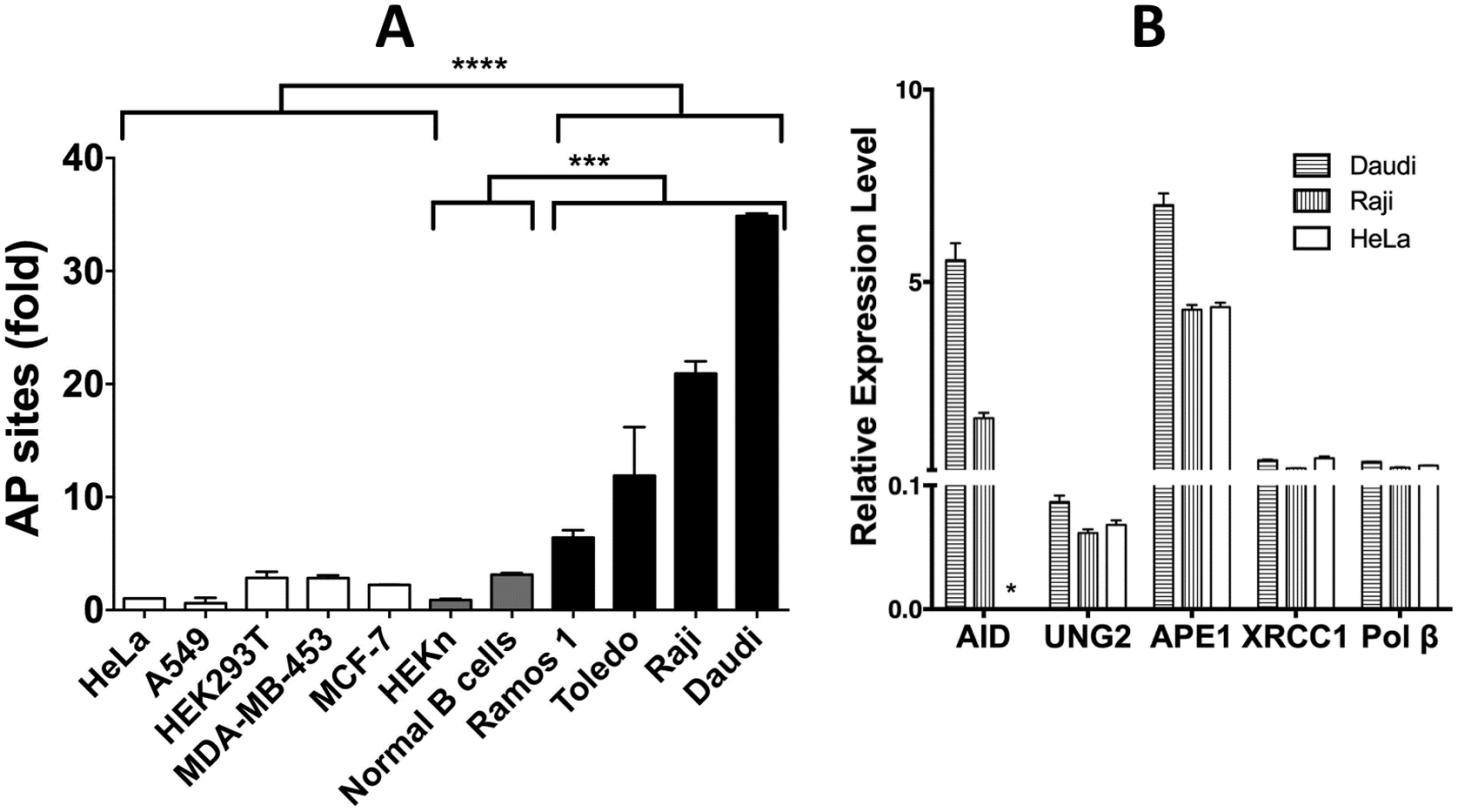
Levels of genomic AP sites and expression genes responsible for the creation and repair of AP sites. (A) Relative levels of AP sites in the DNA of B-NHL and non-B-NHL cells. The level of AP sites of each cell line is shown relative to the level in HeLa cells (set to 1). **** is P-value <0.0001, and *** is P-value <0.0005. In all cases, the mean and standard deviations are shown. (B) The expression of different genes in Daudi, Raji and HeLa cells relative to the β actin gene set at 100. “*” denotes undetectable expression.

Abasic sites in the genomic DNA from a number of different human cancer cell lines and two types of normal primary human cells were quantified using a previously described chemical, AA3, which links to AP sites in DNA (Fig 1A and 1B; [24]), and the results were normalized to the number of AP sites in the HeLa genome. It should be noted that AA3 reacts with intact as well as cleaved AP sites, but not reduced AP sites [24]. The four B-NHL lines (Ramos 1, Toledo, Raji and Daudi) express AID at high levels [17] and have about six- to 35-fold higher levels of AP sites in their genomes compared to HeLa (Fig 2A). In contrast, all the non-B cell lines (A549, HEK293T, MDA-MB-453 and MCF-7) showed undetectable levels of AID expression (S2 Fig) and lower levels of AP sites compared to the B-NHL lines (Fig 2A). While the low levels of AP sites in A549, HEK293T and MCF-7 cells are not unexpected, it is somewhat surprising that MDA-MB-453 cells also contain low levels of AP sites. This latter cell line expresses a DNA-cytosine deaminase related to AID, APOBEC3B, and reportedly contains an excess of uracil in its genome [26]. Therefore, overexpression of a DNA-cytosine deaminase may not be sufficient for the accumulation of AP sites in the genome.

We previously showed that, in contrast to B-NHL cancers, normal B cells from murine spleen or human tonsils do not express AID at high levels or accumulate uracils in their genomes [17]. This suggested that normal B cells may contain fewer AP sites than the B-NHLs. Quantification of AP sites in the genomes of B cells from normal human volunteers showed that they had slightly higher level of AP sites than HeLa cells, but lower than the three B-NHL lines (Fig 2A). Additionally, neonatal human keratinocytes (HEKn) cells also have AP site levels comparable to non-B cell lines. A comparison of AP site levels in the B-NHL lines with non-B cancers or normal cells showed that differences between these groups of cells were statistically quite significant (Fig 2A). These results show that cell lines derived from cancers that express AID at high levels accumulate more AP sites than normal B cells and non-B cancer lines.

As the AP sites in B-NHL genomes are created by the action of UNG2, but are repaired by the BER pathway, lower expression of BER enzymes in B-NHL cells may cause AP sites to accumulate in DNA. To test this possibility, we compared the levels of expression of AID, UNG2, APE-1, XRCC1 and Pol β genes in Daudi and Raji cells with expression in HeLa cells (Fig 2B). While the expression of AID gene was undetectable in HeLa cells (Fig 2B and S2 Fig), the other genes were expressed in HeLa cells and the two B-NHL cell lines at comparable levels (Fig 2B). Therefore, the presence of higher levels of AP sites in Daudi and Raji cells (Fig 2A) cannot be explained by lower expression of genes responsible for BER.

### Sensitivity of B cell lymphoma cell lines to AA3

**Fig 3:**
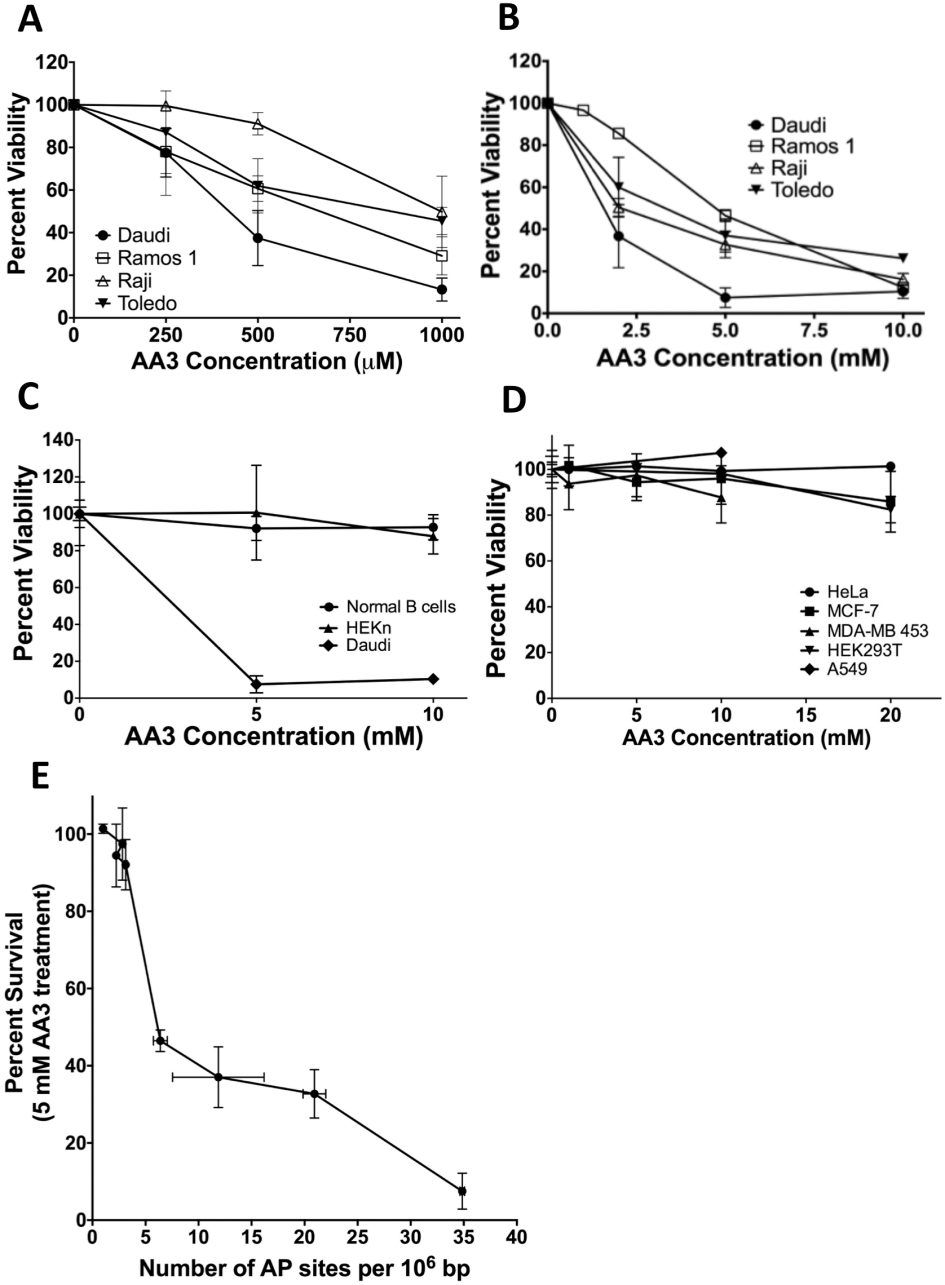
Sensitivities of different cells to AA3. (A) Killing of B-NHL cells by micromolar concentrations of AA3. (B) Killing of B-NHL cells by millimolar concentrations of AA3. (C) Comparison of AA3 sensitivities of Daudi cells, normal human B cells and primary human keratinocytes (HEKn). (D) Comparison of AA3 sensitivity of Daudi cells with non-B cell lines HeLa, MCF-7, MDA-MB 453, A549 and HEK293T. The data in panel A is from triplicates, while the data in the remaining panels is from six replicates. (E) Correlation between AP sites in B-NHL and other cell lines and killing by treatment with 5 mM AA3. These data are from Fig 2, and parts B and D of this figure. In all cases, the mean and standard deviations are shown.

When AA3 reacts with AP sites in DNA forming a stable covalent adduct, it inhibits the first step in the repair of AP sites, DNA cleavage by an AP endonuclease [24]. This suggested that AA3 could block repair of AP sites in B cells resulting in the inhibition of DNA replication and cell death (S1 Fig). To test this, four B-NHL cell lines were treated with AA3 at different concentrations for 24 hours and the cell viability was determined using the trypan blue exclusion assay. The results are shown in Fig 3A and 3B.

All four cell lines were sensitive to sub-millimolar concentrations of AA3 and at 1 mM, AA3 reduced cell viability by about 50% to 80% (Fig 3A). Greater killing was observed in the millimolar range of concentrations and at 10 mM the viability of all the cell lines was reduced below 30% (Fig 3B). Daudi, a Burkitt lymphoma-derived cell line, was consistently most sensitive to AA3 than the other lines and its viability was typically reduced to about 10% at 2 mM AA3 (Fig 3B). For this reason, it was used as a positive control in subsequent experiments.

### Lack of sensitivity of normal cells and non-B cancer lines to AA3

To determine whether normal B cells were sensitive to AA3, B lymphocytes were isolated from the blood of normal volunteers [27] and stimulated to divide for three days using IL-4 and anti-CD40 antibody [28]. These cells and the HEKn cells were treated with 5 or 10 mM AA3 for 24 hrs and cell viability was determined. While neither type of normal cells was significantly killed by AA3, Daudi cells treated in parallel cultures were highly sensitive to the chemical (Fig 3C). Next, we tested four non-B cancer lines and one non-B immortalized cell line for sensitivity to AA3. None of these cells were very sensitive to the chemical (Fig 3D). At the highest concentration of AA3 tested, 20 mM, there was slight sensitivity to the drug, but the reduction in viability was only 10–15% (Fig 3D). This relative lack of sensitivity of these cells to AA3 is consistent with the low levels of AP sites found in their DNA (Fig 2A).

There was an inverse relationship between the percent viability of different human cell lines following 24 hr treatment with AA3 and the genomic AP site levels (Fig 3E). The AA3 concentration of 5 mM was chosen for this comparison because at this concentration some cell killing was seen for many cell lines, but the drug did not show its maximal effect (Fig 3B and 3D). The correlation was strong (Spearman rank coefficient = -0.98; two-tailed P = 0.0004), suggesting that there is a very strong monotonic relationship between the susceptibility of a cell line to killing by AA3 and the level of AP site in its genome.

### AA3 reacts with abasic sites in genomic DNA *in vivo*

**Fig 4:**
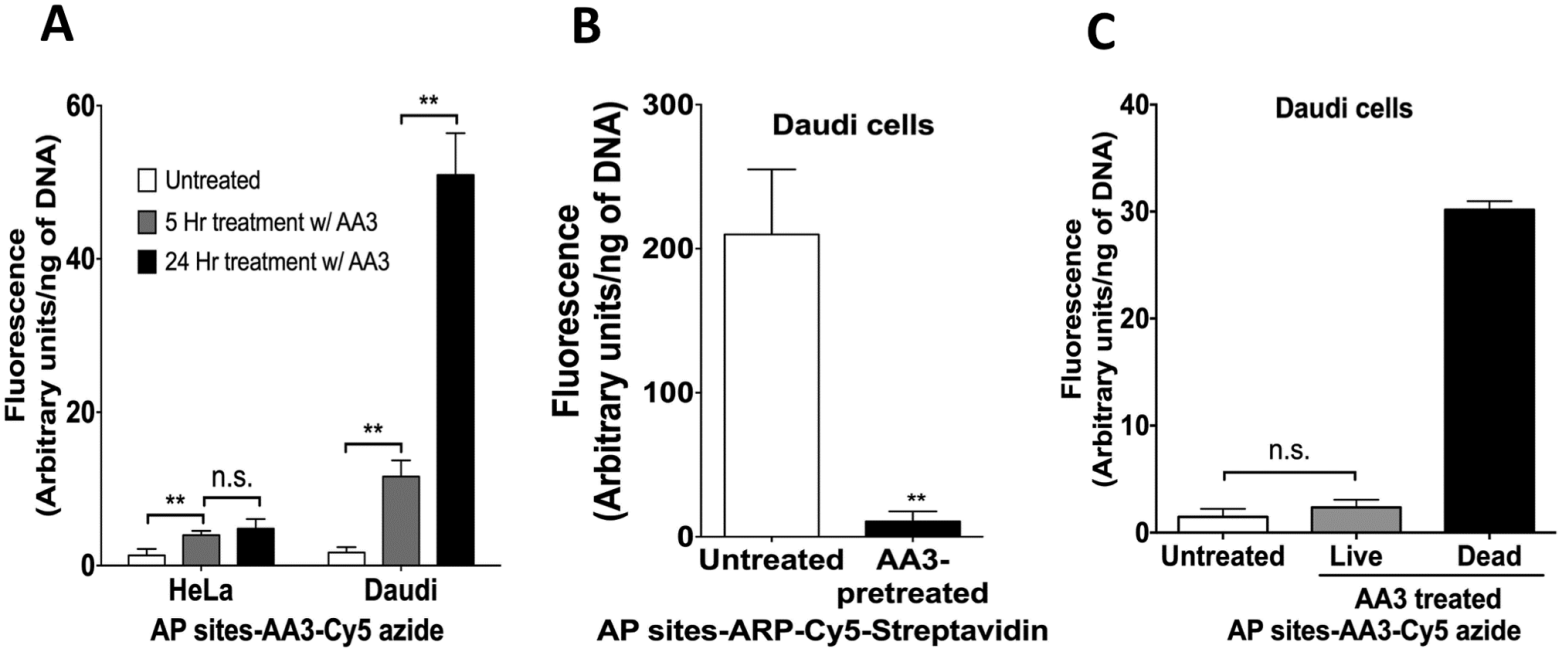
Covalent binding of AA3 to genomic DNA *in vivo*. Fluorescence intensity due to Cy5 bound to genomic DNA is shown. (A) Comparison of labeling of HeLa and Daudi cell DNA with AA3. The cells were treated with AA3 for indicated lengths of time and this was followed by DNA extraction and reaction with Cy5 azide. (B) Comparison of labeling of DNA from untreated cells with cells pretreated with AA3. Genomic DNA was isolated, labeled with ARP and bound to Cy5-Streptavidin. (C) Comparison of binding of AA3 to genomic DNA in dead or dying cells with live cells. Cells were treated with AA3, dead cells were separated from live cells, the genomic DNA was extracted and reacted with Cy5 azide. The data are from six replicates, and the mean and standard deviation are shown. ** represents P-value <0.005, n.s. is not significant.

The alkyne functionality within AA3 can be used to link it with a fluorescent label through click chemistry [24]. We used this property to determine whether or not AA3 links covalently to genomic DNA when Daudi cells are treated with this chemical. HeLa or Daudi cells were treated with AA3 for 5 or 24 hrs and genomic DNA was extracted from the cells. This DNA was reacted with Cy5 azide and Cy5 fluorescence bound to DNA was quantified (Fig 4A).

There was a small, but significant, increase in fluorescence linked to HeLa DNA following 5 hr treatment, but little additional increase was seen when the AA3 treatment was extended to 24 hrs (Fig 4A). Compared to HeLa DNA, the increase in fluorescence intensity linked to Daudi DNA was much greater following both 5 and 24 hrs of AA3 treatment (Fig 4A). Daudi cells had respectively three- and eleven-times as much AA3 linked to its genome as HeLa cells following 5 or 24 hr treatment. These results are consistent with the higher levels of AP sites in extracted Daudi DNA compared to HeLa DNA (Fig 2A) and suggest that AA3 reacts with AP sites in DNA *in vivo*.

We used the chemical ARP, which also links to AP sites (Fig 1; [29, 30]), to determine whether AA3 reacts with all available AP sites in the Daudi genome. Following treatment of Daudi cells with AA3, cellular DNA was extracted and labeled with ARP. When the amounts of AP site-ARP adducts were quantified using Cy5-Streptavidin, DNA from AA3 treated cells showed much lower levels of Cy5 fluorescence than untreated cells (Fig 4B). This shows that at the concentrations used, 5 mM, AA3 links to nearly all available aldehydic lesions in Daudi genomic DNA.

As AA3 kills Daudi cells, we also wished to determine whether there was a difference in the amount of AA3 bound to DNA of cells killed by AA3 compared to the living cells. Following a 5 hr treatment of Daudi cells with 2 mM AA3 (~50% killing), the dead cells were separated from the viable cells using an apoptotic cell isolation kit. DNA was isolated from both the cell populations, reacted with Cy5 azide and the fluorescence was quantified. As expected, Cy5 azide readily reacted with DNA from dead cells showing the presence of covalently linked AA3 in its DNA (Fig 4C). Surprisingly, DNA from non-apoptotic Daudi cells showed about the same fluorescence intensity as the untreated cells (Fig 4C). This result was reproducible (S3 Fig) and suggests that the cells that are not killed by AA3 either contain very few AP sites or are able to repair most AP-AA3 DNA adducts. The Daudi cell population is likely to contain a broad distribution of abilities to create and repair AP sites, and a fraction of the cells that contain low levels of AP sites (e.g. due to lower AID expression) may be able to completely repair those AP sites or any AA3-DNA adducts.

### AA3 causes strand breaks in DNA

It is well established that a variety of DNA modifications including AP sites inhibit replicative DNA polymerases [31], it seemed likely that AA3-AP site adducts would also block DNA replication creating strand breaks. Presumably, this would result in the collapse of the replication fork and cell death (S1 Fig). To test whether or not AA3 causes strand breaks, Daudi cells were treated with AA3 and then stained with anti-γH2AX antibodies.

**Fig 5:**
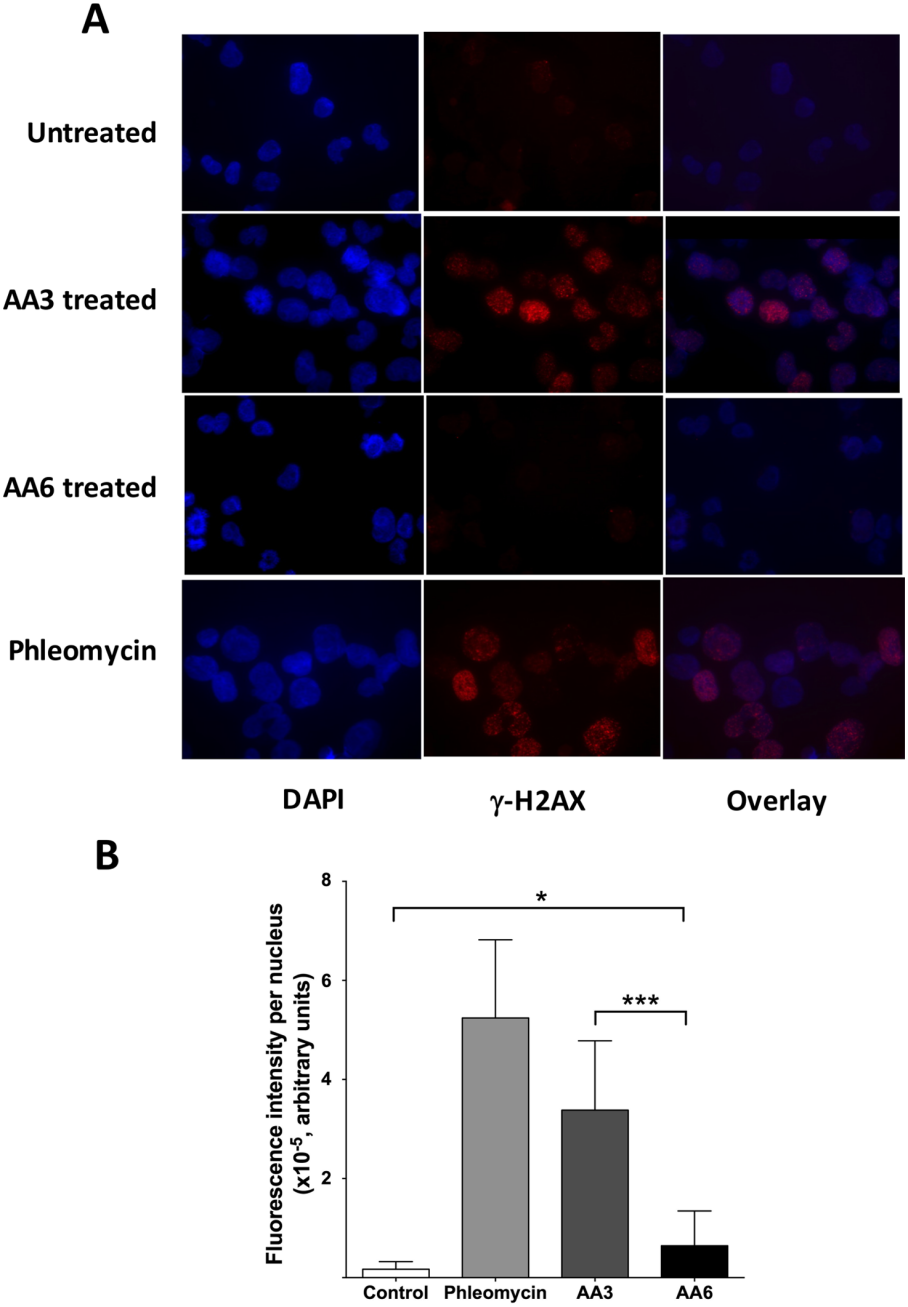
Detection of γ-H2AX in Daudi cells following AA3 treatment. (A) Daudi cells were stained with anti-γ-H2AX antibodies and visualized using immunofluorescence. Untreated cells are compared with AA3- or AA6-treated, or Phleomycin-treated cells. Representative images of cells stained with DAPI (blue), anti-γ-H2AX antibodies labeled with Cy3 (red) and the overlay of the two images are shown. (B) Quantification of γ-H2AX nuclear fluorescence intensity. The total number of cells used for quantification- Untreated, 74; AA6-treated, 70; AA3-treated, 184 and Phleomycin-treated, 122. Mean intensity and standard deviation for each cell type is shown.

While untreated Daudi cells showed few nuclear γH2AX foci, treatment of cells with AA3 or Phleomycin, an analog of bleomycin [32], resulted in a large γH2AX signal (Fig 5A). The signal was concentrated as foci in most nuclei, but many nuclei showed a dispersed fluorescence signal (Fig 5A and S4 Fig). For this reason, we determined the total fluorescence intensity per nucleus instead of counting γH2AX foci. This quantification showed that AA3 treatment resulted in a ~20-fold increase in the γH2AX signal compared to untreated cells (Fig 5B). These results show that AA3 causes extensive breakage of DNA strands in the Daudi genome.

### AA3 cytotoxicity is cell cycle-dependent

As mentioned above, the lethal effects of AA3 are likely to be due to inhibition of replication by AA3-DNA adducts. Thus B-NHL cells that enter the S phase should be more susceptible to killing by AA3 than those that do not replicate. To test this idea, we arrested Daudi cells in the G1 phase by treating them with mimosine. A 24 hr treatment of cells with mimosine increased the number of cells in the G1 phase from 21% to 72% and release of the mimosine block for four hours increased the number of cells in both S and G2 phases (S5 Fig). Untreated cells, cells continuously treated with mimosine and cells treated with mimosine and then released into the S phase were all treated with AA3 for five hrs and cell viability was determined.

**Fig 6:**
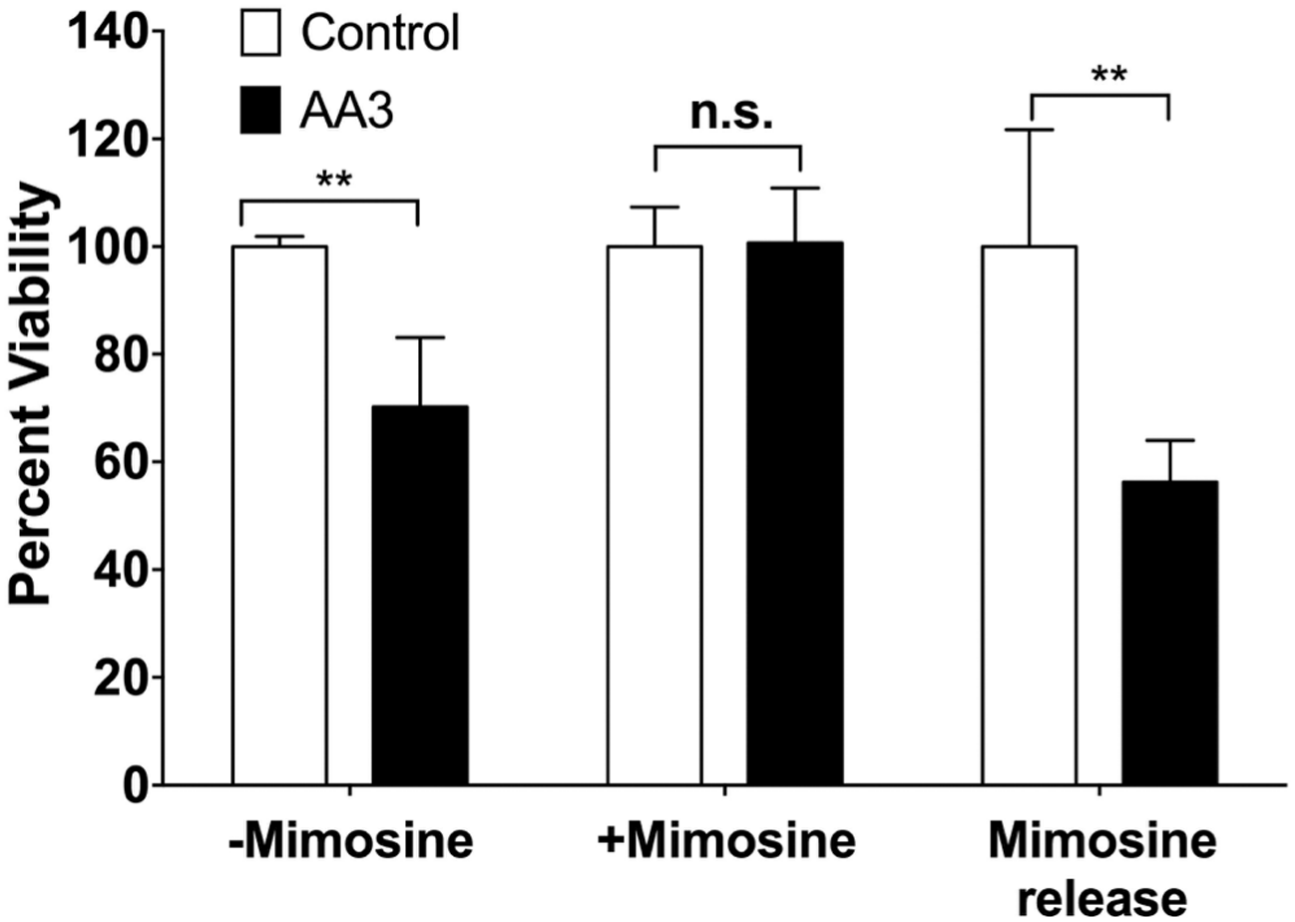
Insensitivity of G1-arrested Daudi cells to AA3. Daudi cells grown in normal growth media (-Mimosine), cells blocked in G1 using Mimosine treatment (+Mimosine) and cells released from Mimosine block (Mimosine release) were incubated with AA3 and the cell viability was determined. The numbers were normalized to the viability of cells without AA3 treatment (set to 100). The data are shown as the mean and standard deviation from six replicates (** represents P-value <0.005, n.s. is not significant).

Cells blocked in G1 were insensitive to AA3 (Fig 6). In contrast, the cells that were released from the mimosine block regained their sensitivity to AA3 (Fig 6). These results show that AA3 kills B-NHL cells that are replicating.

### Inhibition of APE-1 activity is not sufficient for killing B-NHL cells

Methoxyamine (MX; Fig 1B) reacts with AP sites in a manner similar to AA3 and inhibits APE-1 [33]. It has been used to increase the effectiveness of chemotherapeutic agents that cause DNA damage that is repaired through BER [33–38]. However, unlike AA3, MX has not been shown to kill any cancer cells on its own. ARP (Fig 1B) is also known to react with AP sites [29, 30], but has also not been shown to kill cancer cells. We tested the possibility that MX and ARP may kill B-NHL cells.

**Fig 7:**
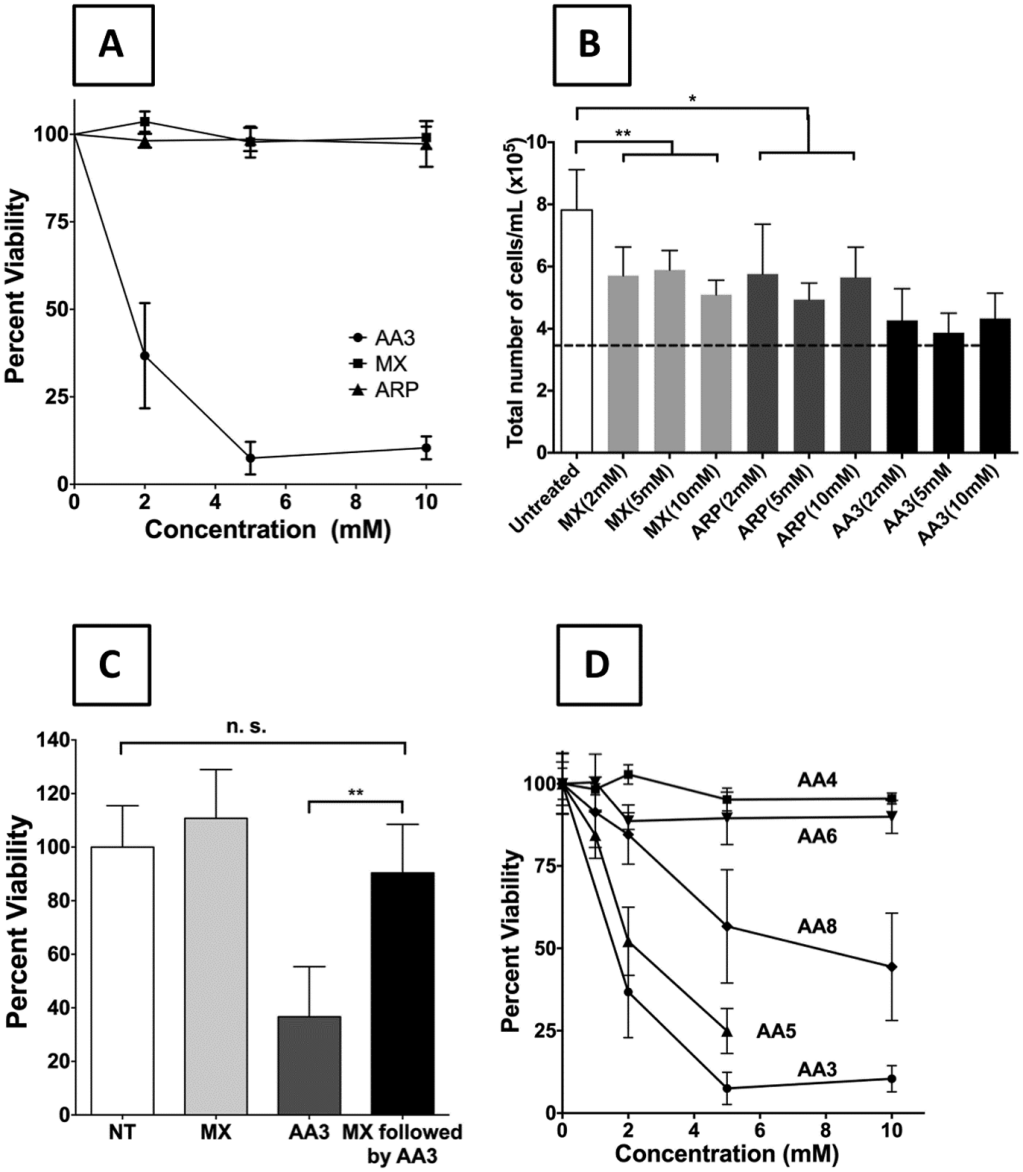
Comparison of the sensitivity of a B-NHL cell line to different alkoxyamines. (A) Comparison of sensitivity of Daudi cells to MX, ARP or AA3. (B) Reduction in cell growth following treatment of Daudi cells with MX, ARP and AA3. Cells were treated for 24 hr at indicated concentrations of chemicals. Horizontal dotted line represents the starting density of cells. “*” is P-value of <0.05 and “**” is P-value of <0.01. (C) Suppression of AA3 cytotoxicity for Daudi cells by pre-treatment of the cells with MX. (D) Comparison of cytotoxicities of different analogs of AA3 for Daudi cells. In all cases, the viability of untreated cells (NT) is set at 100% and the mean and standard deviation of six replicates are shown (** represents P-value <0.005, n.s. is not significant).

Neither MX nor ARP killed Daudi cells (Fig 7A). At the maximum concentrations tested, 10 mM, AA3 reduced the viability of Daudi cells to ~10% while MX or ARP showed no significant effect on cell viability (Fig 7A). These two chemicals also did not significantly kill other B-NHL cells at 10 mM or HeLa cells at 20 mM (S6 Fig). However, both the chemicals significantly inhibited Daudi cell growth (Fig 7B) suggesting that MX and ARP are cytostatic, but cytotoxic for B-NHL cells.

It seemed possible that the lack of sensitivity of Daudi cells and other B-NHLs to MX or ARP may be because these chemicals are not efficiently taken up by the cells or are actively exported by them. To test this possibility, we pre-treated Daudi cells with MX followed by AA3 treatment, and determined the effects of this sequential treatment on cell viability. The results show that MX pre-treatment suppresses the toxicity of AA3 (Fig 7C) and strongly suggest that both MX and AA3 react with the same cellular targets, presumably AP sites in DNA.

**Fig 8:**
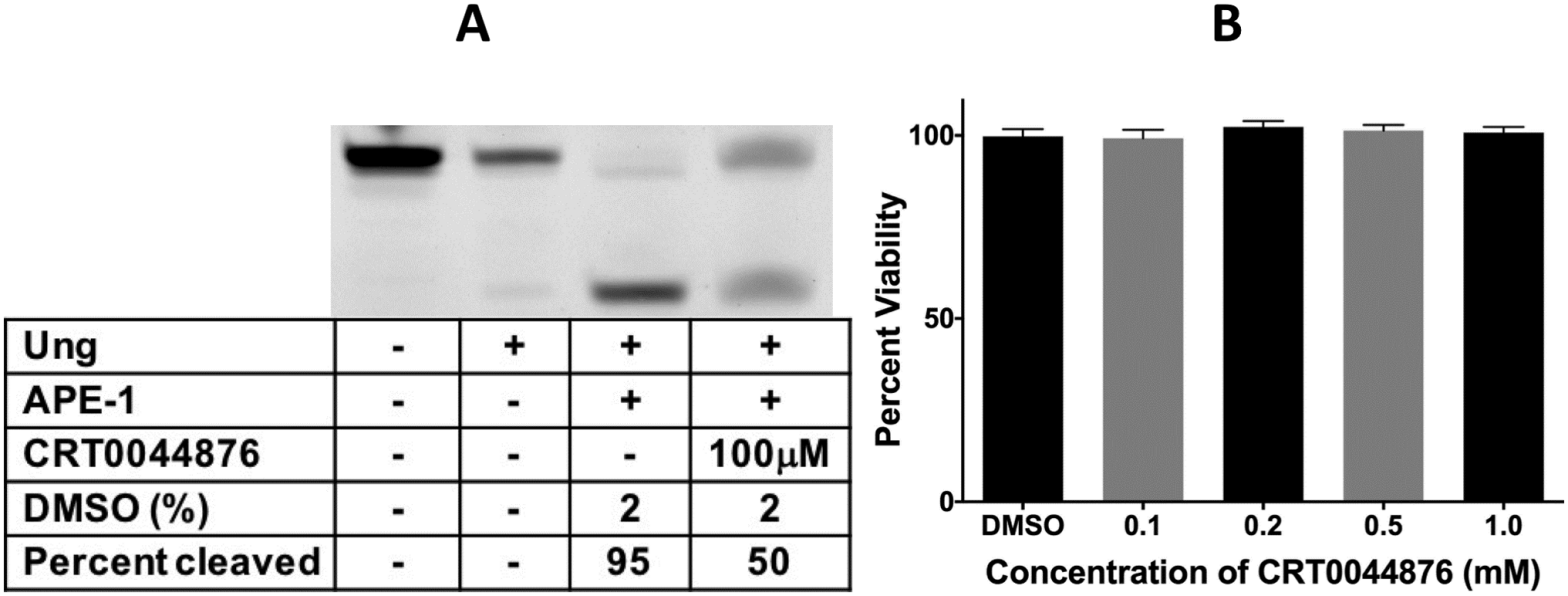
Effects of CRT0044876 on APE-1 and Raji cells. (A) A synthetic oligomer containing a uracil was treated with Ung to create an AP site and the AP site cleaved by APE-1 in the presence or absence of the inhibitor CRT0044876 (100 μM). (B) Raji cells were treated with different concentrations of CRT0044876 dissolved in DMSO and the viability of the cells after 24 hr treatment is presented. “DMSO” represents viability of cells following treatment with 1% DMSO alone.

A number of inhibitors of APE-1 have been described that do not contain the alkoxyamine or alkyne functionalities [39–46]. We tested one of them, CRT0044876 [42], for its ability kill Raji cells. CRT0044876 inhibits APE-1 at 100 μM (Fig 8A), but is not toxic to Raji cells even at 1 mM (Fig 8B). At this concentration AA3 causes greater than 50% reduction in cell viability (Fig 3A). This confirms that the ability to inhibit APE-1 is not sufficient to explain the killing of B-NHL cells by AA3.

### Alkyne functionality of AA3 is important for its toxicity

The lack of toxicity of MX and ARP for B-NHL cells suggested that the alkoxyamine functionality within AA3 was not sufficient for its biological action. As the only other functional group within AA3 is the weakly reactive alkyne group connected to the alkoxyamine through a methylene bridge (Fig 1B), we synthesized variants of this functional group and tested them for their toxicity towards Daudi cells. Specifically, we synthesized and tested molecules in which the alkyne functionality was replaced with a methyl group (AA4) or an azide (AA6; Fig 1B). We also synthesized a molecule in which the alkyne was separated from the oxygen by an additional methylene bridge (AA5) or was internal to the molecule (AA8; Fig 1B). The ability of these new alkoxyamines to react with AP sites was confirmed using an inhibition assay [24]. Specifically, prior incubation of AP site containing DNA oligomer with AA4, AA5, AA6 or AA8 inhibits the reaction of the AP sites with SS-ARP (S7 to S10 Figs).

Remarkably, both the compounds lacking the alkyne functionality, AA4 and AA6, were non-toxic to Daudi cells (Fig 7D). To test whether these non-toxic alkoxyamines cause strand breaks in Daudi genome, we compared the abilities of AA3 and AA6 to cause γH2AX foci in Daudi nuclei (Fig 5). Although the AA6 treated Daudi cells did show slightly higher γH2AX signal compared to untreated cells, the AA3 treated cells had five times the signal seen with AA6 (Fig 5B). Therefore, AA6 is much less effective at causing DNA strand breaks than AA3.

In contrast to AA4 or AA6, compounds AA5 and AA8 contain the alkyne functionality (Fig 1B). These alkoxyamines killed Daudi cells, but less effectively than AA3 (Fig 7D and S11 Fig). These results suggest that the normally bioorthogonal alkyne functional group [47, 48] is required for the toxicity of these alkoxyamines towards B-NHL cells and their effectiveness is modulated by the distance between alkoxyamine and alkyne groups, and whether the alkyne functional group is internal to the molecule or at its terminus.

## Discussion

We showed here that some alkoxyamines that react with AP sites in DNA selectively kill cells derived from B-cell cancers. In particular, three such chemicals, AA3, AA5 and AA8, kill cells from B-NHL cancers (Figs 3A, 3B and 7C). The non-B cancer cells are resistant to AA3 (Fig 3D) and this drug resistance correlates with low levels of AID expression (S2 Fig), and AP sites (Fig 3E). Normal B lymphocytes and primary keratinocytes also have few AP sites in their genomes (Fig 2A) and are insensitive to AA3 (Fig 3C). When the B-NHL-derived cell line, Daudi is treated with this chemical, it links covalently to the genomic DNA (Fig 4A) and is found selectively in the DNA of dead or dying cells (Fig 4C). Additionally, AA3 causes DNA strand breaks in Daudi genome, and cell killing by AA3 is dependent on the ability of the cells to replicate their DNA.

These observations are consistent with a mode of action of AA3 in which the chemical links to AP sites in DNA inhibiting cleavage of the sites by APE-1 [24], resulting in a lethal replication block (S1 Fig). However, a known inhibitor of APE-1, CRT0044876, does not kill a B-NHL cell line at concentrations at or above which it inhibits APE-1 (Fig 8B). Furthermore, we previously showed that MX, and to a lesser extent, ARP also inhibits the ability APE-1 to cleave AP sites [24], but neither chemical kills Daudi (Fig 7A) and other B-NHL cells (S6 Fig). Both MX and ARP inhibited growth of Daudi cells (Fig 7B), which is consistent with previous observations that a knockdown of APE-1 in an ovarian cancer cell line inhibits cell growth without an increase in apoptosis [49]. In other words, the ability of alkoxyamines to react with AP sites and inhibit APE-1 slows cell growth, but does not result in cell death. Additionally, neither AA4 nor AA6 kills Daudi cells (Fig 7D) suggesting that a more complex mechanism causes cell death by alkoxyamines. Hence the alkyne functionality in AA3, AA5 and AA8 may play a key role in their toxicity.

For example, it is formally possible that following the linking of AA3, AA5 or AA8 at AP sites, the alkyne functional group in these molecules binds and inhibits one or more enzymes downstream APE-1 in the BER pathway. The presence of the carbon-carbon triple bond makes the molecules AA3, AA5 and AA8 more rigid than AA4 and this may provide a structural reason for AA4 inactivity (Fig 7D). However, the azide group in AA6 is also rigid and despite this AA6 does not kill Daudi cells (Fig 7D). Additionally, it is unlikely that the terminal alkyne in AA3 and AA5 fits into a hydrophobic pocket of some protein necessary for the survival of B-NHL cells. If that were so, AA8 would have been expected to be non-toxic to these cells. However, this is not the case (Fig 7D) and hence the alkyne group is unlikely to play a structural role in cell killing.

Another possibility is that the alkyne functionality within AA3 and AA5 could react with adducts in DNA or proteins bound to DNA. Such reactions could create intra- or inter-strand crosslinks in DNA, or protein-DNA crosslinks that are difficult to repair and this causes cell death. In effect, AA3, AA5 and AA8 may be acting as reactive bifunctional agents similar to nitrogen mustards or cis-platin.

The principal problem with this hypothesis is that alkynes are not highly reactive and hence are thought to be “bioorthogonal”, i.e. unreactive towards biomolecules [47, 48]. Thus, the alkynes in AA3, AA5 and AA8 may have to be activated by a more strongly reactive molecule such as a radical [50] in order for them to react with cellular components. Alternately, a highly reactive cellular biomolecule may directly link to the alkyne creating a DNA adduct that is difficult to repair. There is some precedence for an alkyne group making a chemical more toxic. Propargyl alcohol is lethal to rats and other animals at much lower doses than 1-propanol [51–53]. However, the reasons for the higher toxicity of propargyl alcohol is unknown and it is unclear whether this is related to the toxicity of the alkoxyamines with alkyne functionality studied here.

In summary, we have found a novel class of compounds that covalently link to AP sites in DNA and specifically kill immortalized cells obtained from many B cell lymphomas at concentrations of hundreds of micromolar. Although these high concentrations are of some concern because of the possibility of side reactions, the AA3 family of compounds are attractive prototypes for anticancer agents because of their high selectivity- they do not kill normal B cells or non-B cancer cells even at 10 mM. However, we do not know the pharmacokinetics or toxicity of these chemicals in animal models. While the concentrations at which these chemicals kill the cells are unlikely to be clinically useful, they appear to act through a completely new mechanism. Therefore, elucidating this mechanism of action may suggest strategies to improve the effectiveness of the existing alkoxyamines and/or to synthesize analogs of these alkoxyamines that would selectively kill cancer cells at submicromolar concentrations.

## Supporting information

S1 Fig A model for cell killing by AA3. (PDF)

S2 Fig AID expression levels in B-NHL and non-B cell lines. (PDF)

S3 Fig Comparison of the levels of Cy5 fluorescence bound to DNA of dead and living Daudi cells following the treatment of cells with AA3 followed by DNA extraction and reaction with Cy5 azide. (PDF)

S4 Fig Visualization of γ-H2AX in Daudi nuclei following AA3 treatment at 60X magnification using Nikon E800 fluorescence microscope. (PDF)

S5 Fig Cell cycle analysis by flow cytometry following mimosine treatment. (PDF)

S6 Fig The lack of cytotoxicity of MX and ARP for B-NHL and HeLa cell lines. (PDF)

S7 Fig AA4 blocks the reaction of ssARP at AP sites in DNA. (PDF)

S8 Fig AA5 blocks the reaction of ARP at AP sites in DNA. (PDF)

S9 Fig AA6 blocks reaction of ARP at AP sites in DNA. (PDF)

S10 Fig AA8 blocks reaction of ARP at AP sites in DNA. (PDF)

S11 Fig Comparison of cell killing ability of AA3 with AA5 and AA8. (PDF)

S1 Table Primers used for RT-PCR. (PDF)
