# Supplementary material for: A novel class of chemicals that react with abasic sites in DNA and specifically kill B cell cancers
Source: PLoS One. 2017 Sep 19;12(9):e0185010. doi: 10.1371/journal.pone.0185010 (PMC5605088; doi:10.1371/journal.pone.0185010)
Supplement: S1 Fig — (PDF) [file pone.0185010.s001.pdf]

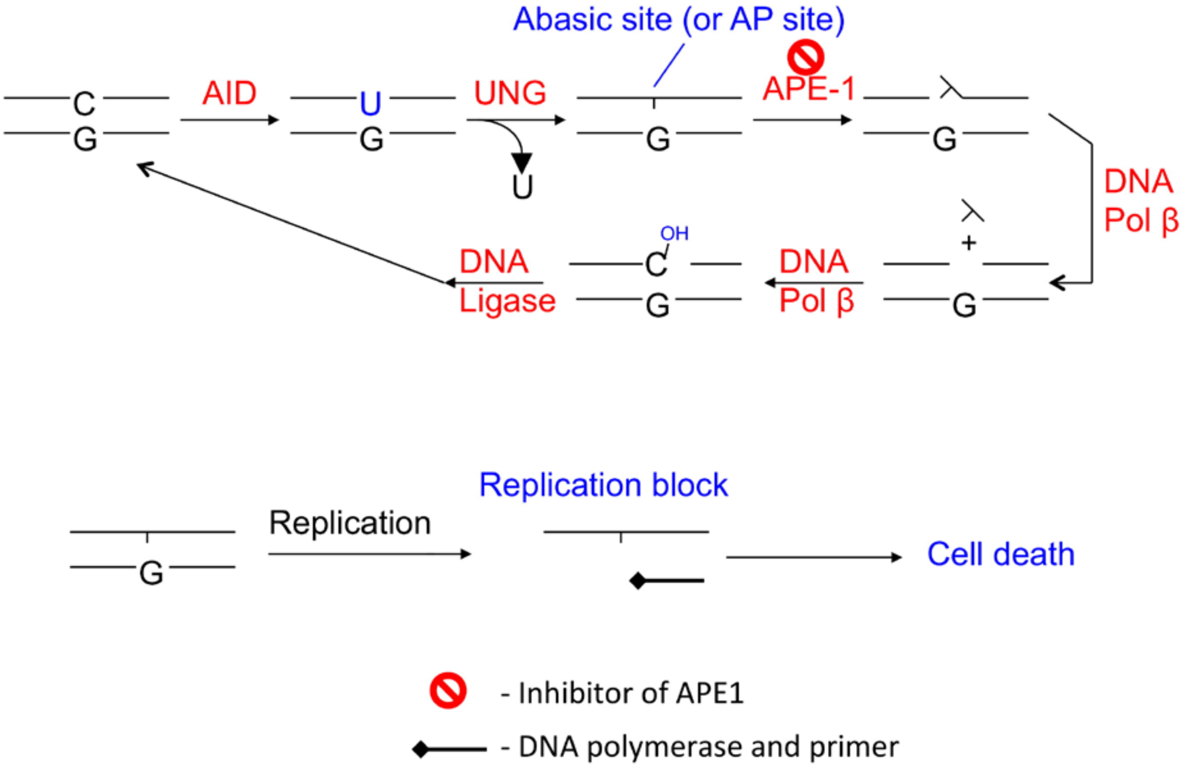

S1 Figure. A model for cell killing by AA3.

Top- Rationale for cell killing by AA3. B-NHL cells overexpress activation-induced deaminase (AID) which converts cytosine into uracil and results in the accumulation of uracils in their genome. These cells also contain uracil-DNA glycosylase (UNG) that removes these uracils generating AP sites. AP sites are further processed by the AP endonuclease (APE-1) and repaired through the actions of DNA polymerase β.

Bottom- AA3 inhibits the activity of APE1 by reacting with AP sites and blocks the progress of replication fork causing cell death.
