# Supplementary material for: A novel class of chemicals that react with abasic sites in DNA and specifically kill B cell cancers
Source: PLoS One. 2017 Sep 19;12(9):e0185010. doi: 10.1371/journal.pone.0185010 (PMC5605088; doi:10.1371/journal.pone.0185010)
Supplement: S2 Fig — (PDF) [file pone.0185010.s002.pdf]

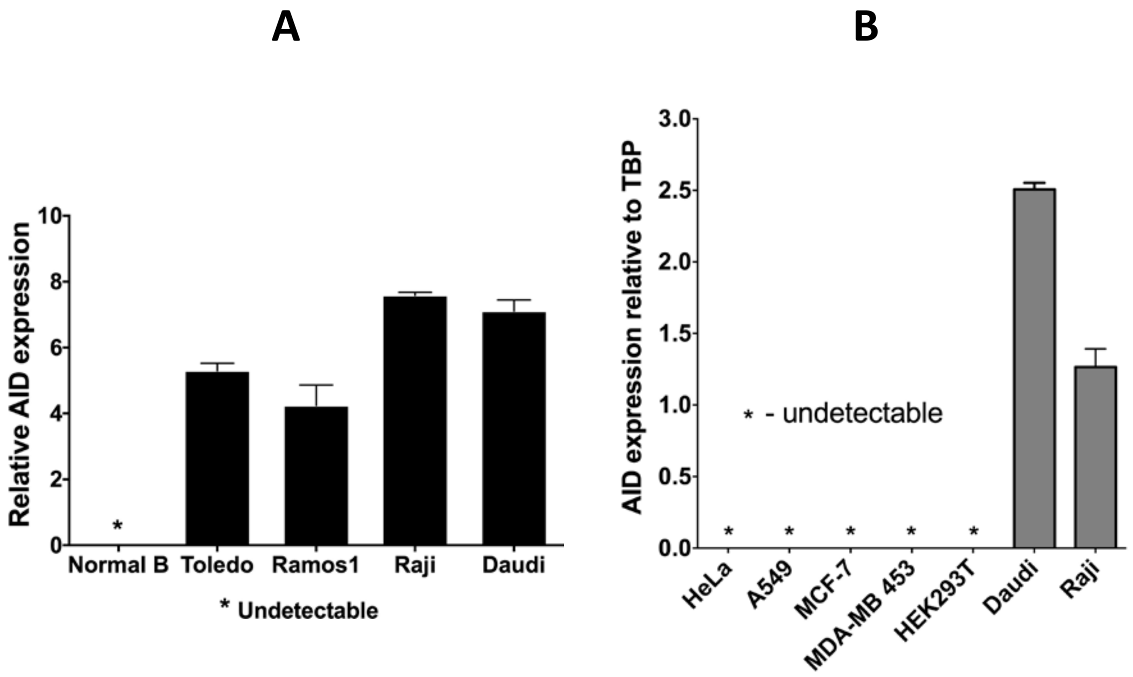

S2 Figure. AID expression levels in B-NHL and non-B cell lines.

A. The levels of AID gene expression were determined using real-time PCR and are shown relative to gene expression levels of GAPDH set at 100. These data are from Shalhout *et al* (2014)[1]. **B.** The levels of AID gene expression were determined using real-time PCR and are shown relative to gene expression levels of TBP. The asterisk (*) indicates undetectable AID expression.

[1] Shalhout, S., et al. (2014). "Genomic uracil homeostasis during normal B cell maturation and loss of this balance during B cell cancer development." Mol Cell Biol **34**(21): 4019-4032.
