# Supplementary material for: A novel class of chemicals that react with abasic sites in DNA and specifically kill B cell cancers
Source: PLoS One. 2017 Sep 19;12(9):e0185010. doi: 10.1371/journal.pone.0185010 (PMC5605088; doi:10.1371/journal.pone.0185010)
Supplement: S3 Fig — (PDF) [file pone.0185010.s003.pdf]

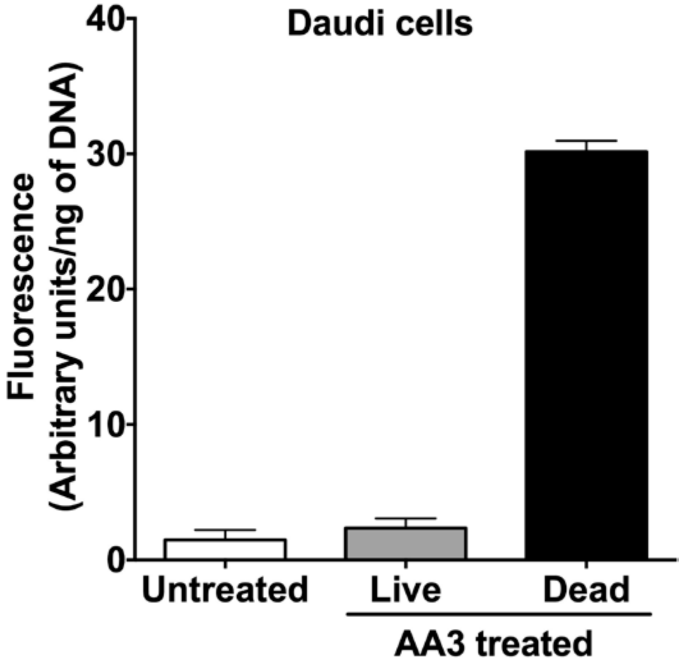

S3 Figure. Comparison of the levels of Cy5 fluorescence bound to DNA of dead and living Daudi cells.

Daudi cells were treated with AA3 for 5 hours and dead cells were isolated from living cells. DNA was extracted from both the cell populations and reacted with Cy5 azide. The normalized fluorescence intensity for the different DNA samples is shown.
