# Supplementary material for: A novel class of chemicals that react with abasic sites in DNA and specifically kill B cell cancers
Source: PLoS One. 2017 Sep 19;12(9):e0185010. doi: 10.1371/journal.pone.0185010 (PMC5605088; doi:10.1371/journal.pone.0185010)
Supplement: S4 Fig — (PDF) [file pone.0185010.s004.pdf]

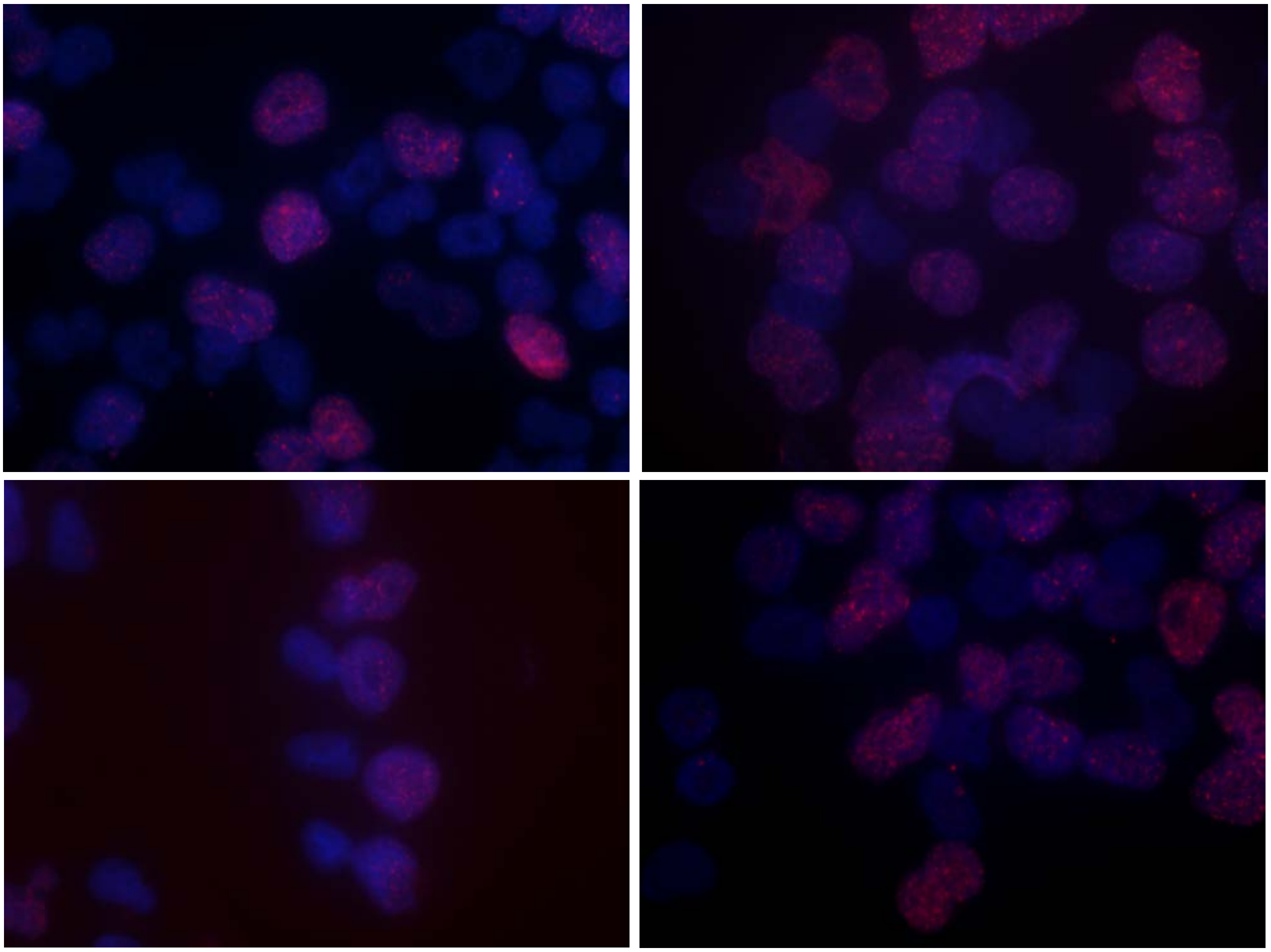

S4 Figure. Visualization of γ-H2AX in Daudi nuclei following AA3 treatment at 60X magnification using Nikon E800 fluorescence microscope.

Representative γ-H2AX immunofluorescence images of Daudi cells after 5-hour AA3 treatment are shown. The cells were stained with anti γ-H2AX antibody (red) and DAPI (blue).
