# Supplementary material for: A novel class of chemicals that react with abasic sites in DNA and specifically kill B cell cancers
Source: PLoS One. 2017 Sep 19;12(9):e0185010. doi: 10.1371/journal.pone.0185010 (PMC5605088; doi:10.1371/journal.pone.0185010)
Supplement: S5 Fig — (PDF) [file pone.0185010.s005.pdf]

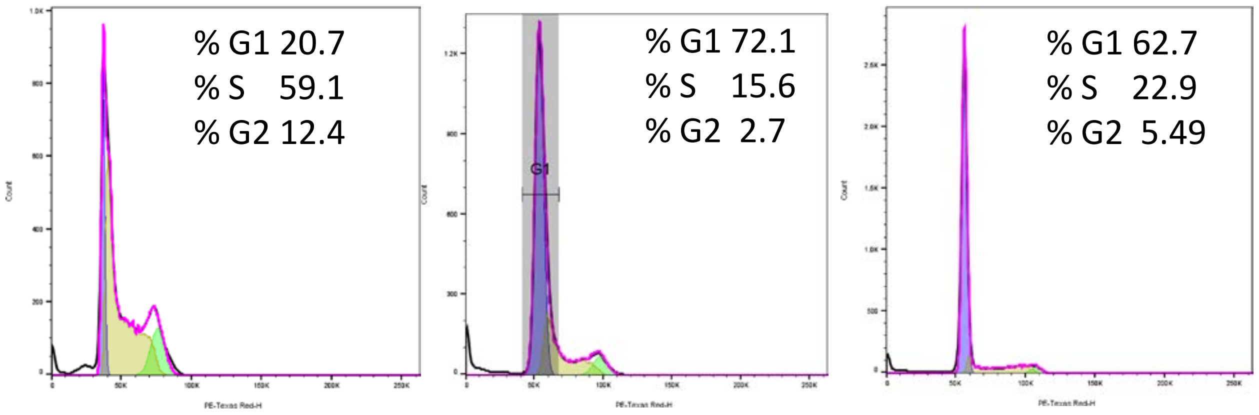

Control　　Mimosine treated　　Mimosine removed

S5 Figure. Cell cycle analysis by flow cytometry following mimosine treatment.

Left- Untreated Daudi cells (control). Following a 24 hr mimosine treatment, the cells were divided into two parts. One culture was grown in medium containing Mimosine for 4 hours (Center; Mimosine treated). The other culture was grown in normal growth media for the same length of time (Right; Mimosine removed). All the cells were collected, and stained with propidium iodide for flow cytometric analysis.
