# Supplementary material for: A novel class of chemicals that react with abasic sites in DNA and specifically kill B cell cancers
Source: PLoS One. 2017 Sep 19;12(9):e0185010. doi: 10.1371/journal.pone.0185010 (PMC5605088; doi:10.1371/journal.pone.0185010)
Supplement: S6 Fig — (PDF) [file pone.0185010.s006.pdf]

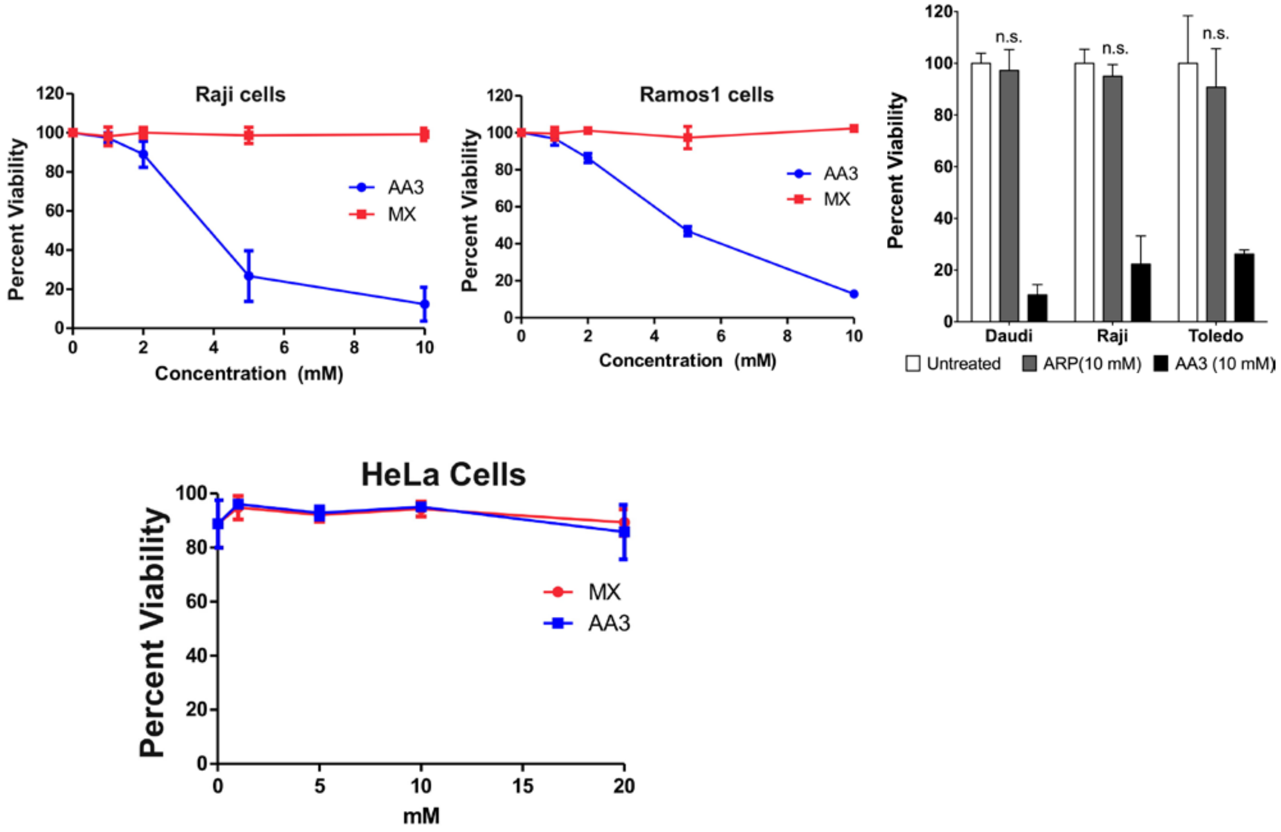


**S6 Figure. The lack of cytotoxicity of MX and ARP for B-NHL and HeLa cell lines.**

The cells were treated with MX, ARP or AA3 for 24 hours, and cell killing was analyzed. The mean and standard deviation are shown in each case (n.s. = not significant).
