# Supplementary material for: A novel class of chemicals that react with abasic sites in DNA and specifically kill B cell cancers
Source: PLoS One. 2017 Sep 19;12(9):e0185010. doi: 10.1371/journal.pone.0185010 (PMC5605088; doi:10.1371/journal.pone.0185010)
Supplement: S7 Fig — (PDF) [file pone.0185010.s007.pdf]

Ung	+	+	+	+	+	+
AA3	-	+	-	-	1st	-
ssARP	-	-	+	-	2nd	2nd
AA4	-	-	-	+	-	1st
Percent Product			85		19	8
	**1**	**2**	**3**	**4**	**5**	**6**

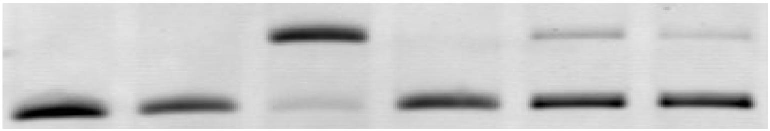

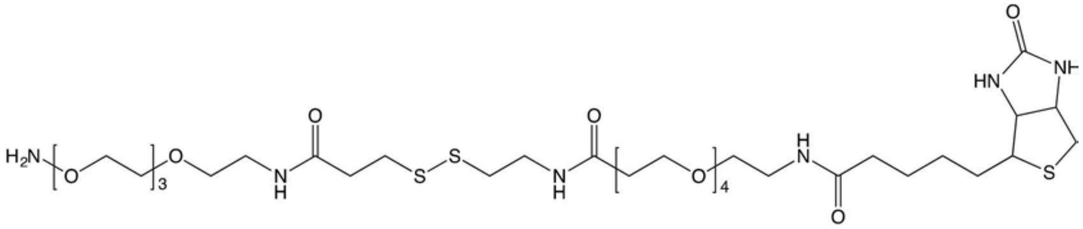

SS-ARP

S7 Figure. AA4 blocks the reaction of ssARP at AP sites in DNA.

An oligonucleotide containing a 6-FAM dye at the 5’ end and an internal uracil was treated with the *E. coli* Ung to create an AP site. The DNA was then treated with the indicated molecules individually or sequentially. The products were separated on a denaturing gel and scanned for fluorescence intensity. ssARP is similar to ARP shown in Fig. 1, but contains a disulfide linkage within the linker between the alkoxyamine and the biotin. Its structure is shown above. It causes mobility shift for the DNA (lane 3). 1st- indicates the chemical that was reacted first with DNA; 2nd- indicates the chemical that was reacted second with DNA. Both AA3 (lane 5) and AA4 (lane 6) block this mobility shift. Both AA3 (lane 2) and AA4 (lane 4) are too small to cause a mobility shift on their own.
