# Supplementary material for: A novel class of chemicals that react with abasic sites in DNA and specifically kill B cell cancers
Source: PLoS One. 2017 Sep 19;12(9):e0185010. doi: 10.1371/journal.pone.0185010 (PMC5605088; doi:10.1371/journal.pone.0185010)
Supplement: S8 Fig — (PDF) [file pone.0185010.s008.pdf]

Ung	-	+	+	+	+	+	+
AA3	-	-	+	-	-	1st	-
ssARP	-	-	-	+	-	2nd	2nd
AA5	-	-	-	-	+	-	1st
Percent Product				94		23	26
	**1**	**2**	**3**	**4**	**5**	**6**	**7**

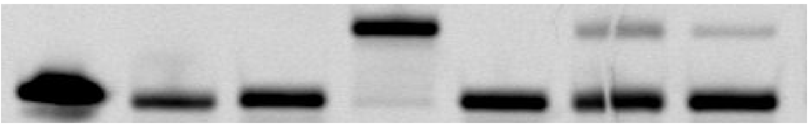

S8 Figure. AA5 blocks the reaction of ARP at AP sites in DNA.

The experimental procedure was the same as described for Supplementary Figure S7.
