# Supplementary material for: A novel class of chemicals that react with abasic sites in DNA and specifically kill B cell cancers
Source: PLoS One. 2017 Sep 19;12(9):e0185010. doi: 10.1371/journal.pone.0185010 (PMC5605088; doi:10.1371/journal.pone.0185010)
Supplement: S10 Fig — (PDF) [file pone.0185010.s010.pdf]

UDG	-	+	+	+	+	+	+
ssARP	-	-	+	-	2nd	-	2nd
AA6	-	-	-	+	1st	-	-
AA8	-	-	-	-	-	+	1st
Percent product	-	-	97	-	7	-	22

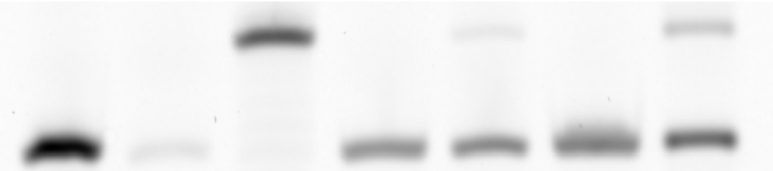

S10 Figure. AA8 blocks reaction of ARP at AP sites in DNA.

The experimental procedure was the same as described for Supplementary Figure S7.
