# Supplementary material for: A novel class of chemicals that react with abasic sites in DNA and specifically kill B cell cancers
Source: PLoS One. 2017 Sep 19;12(9):e0185010. doi: 10.1371/journal.pone.0185010 (PMC5605088; doi:10.1371/journal.pone.0185010)
Supplement: S11 Fig — (PDF) [file pone.0185010.s011.pdf]

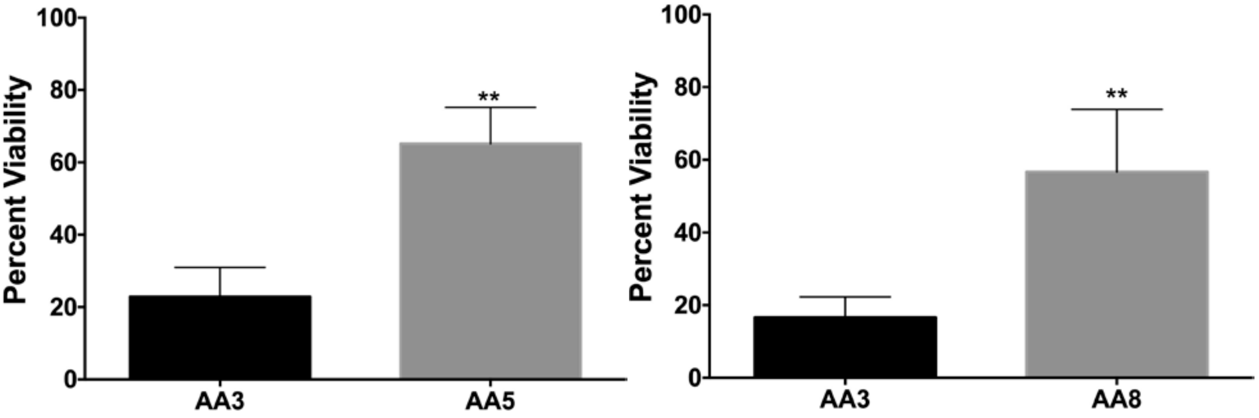

S11 Figure. Comparison of cell killing ability of AA3 with AA5 and AA8

Six replicates of dividing Daudi cell cultures were treated with either AA3 or AA5, and in separate experiment with either AA3 or AA8 at 5 mM. Following 24 hr treatment cell viability was determined by the Trypan Blue assay. The viability was normalized with respect to untreated cells and the percent viability following AA3 treatment was compared with viability following AA5 or AA8 treatment. “**” represents P-value ≤ 0.01.
