# Supplementary material for: A novel class of chemicals that react with abasic sites in DNA and specifically kill B cell cancers
Source: PLoS One. 2017 Sep 19;12(9):e0185010. doi: 10.1371/journal.pone.0185010 (PMC5605088; doi:10.1371/journal.pone.0185010)
Supplement: S1 Table — (PDF) [file pone.0185010.s012.pdf]

Gene	Forward Primer	Reverse Primer
AID	5'-CTGGACTTTGGTTATCTTCG-3'	5'-AAGGTCATGATGGCTATTTG-3'
UNG2	5'-CCTCCTCAGCTCCAGGATGA-3'	5'-TCGCTTCCTGGCGGG-3'
Pol β	5'-GAACACTCTGGGGTTCTCGG-3'	5'-TGTGGATAGCTTGGCTCACG-3'
APE-1	5'-GATCTCGCGAGTAGGGCAAC-3'	5'-TTTGCGGCCGTCTTACTCTT-3'
XRCC1	5'-GACGTTGACATGCCGGAGAT-3'	5'-GAGATGGTCTTCTCGCCTGC-3'
β-ACTIN (ACTB)	5'-TTGCCGACAGGATGCAGAAGGA-3'	5'-AGGTGGACAGCGAGGCCAG-3'

S1 Table. Primers used for RT-PCR
